# Correction: The vascular Na,K-ATPase: clinical implications in stroke, migraine, and hypertension

**DOI:** 10.1042/CS-2022-0796C_COR

**Published:** 2023-12-14

**Keywords:** blood pressure, digoxin, migraine, stroke

The authors of the review article, “The vascular Na,K-ATPase: clinical implications in stroke, migraine, and hypertension” (doi: 10.1042/CS20220796), would like to correct their paper.

The authors identified an error in the last two sentences of the first paragraph in the section “Altering the ouabain-sensitivity of the α2 isoform confers protection against hypertension”. The final two sentences of this first paragraph should read:

“When these two mutations were combined in the knock-in mice, which, in contrast with wild types, had the ouabain-sensitive α1 isoform and ouabain-insensitive α2 isoform, they remained sensitive to develop ACTH-induced hypertension associated with chronic elevation of ouabain [251]. These findings emphasize the crucial role of the ouabain-sensitive isoform in the development of hypertension [7, 252].”

The authors apologise for this error. Mike Ryan (EiC of CS) has approved this correction.

